# A diverticulum of the left ventricular apex manifested by recurrent chest pain

**DOI:** 10.21542/gcsp.2025.52

**Published:** 2025-10-31

**Authors:** Husam Katib, Sarah Alem, Nawfal Alkhafaji, Hamza Yousaf, Manoj Sharma

**Affiliations:** 1 University at Buffalo, Jacobs School of Medicine and Biomedical Sciences, Department of Internal Medicine, Buffalo, NY, USA; 2 Trinity Medical Cardiology, Buffalo, NY, USA

## Abstract

A left ventricular diverticulum at the apex is a rare cardiac anomaly in adults, often detected incidentally due to its asymptomatic nature. Noninvasive imaging modalities, such as echocardiography, provide initial diagnostic clues, but advanced techniques like cardiac magnetic resonance (CMR) imaging or contrast-enhanced computed tomography (CT) are often required for confirmation. This report describes a case of a patient presenting with recurrent chest pain, ultimately diagnosed with a left ventricular apical diverticulum through a stepwise imaging approach.

## Case presentation

A 57-year-old African American woman with a history of dyslipidemia, type 2 diabetes mellitus, and hypertension presented with recurrent substernal chest pain. The episodes occurred unpredictably, lasted up to one minute, and resolved spontaneously without identifiable triggers or alleviating factors. She denied associated symptoms such as shortness of breath, syncope, or palpitations. Her social history included a 10 pack-year smoking history, with cessation 10 years prior, and no family history of congenital or cardiac diseases. On examination, vital signs were normal (blood pressure 128/78 mmHg, heart rate 72 beats/min), and physical findings were unremarkable.

**Figure 1.**
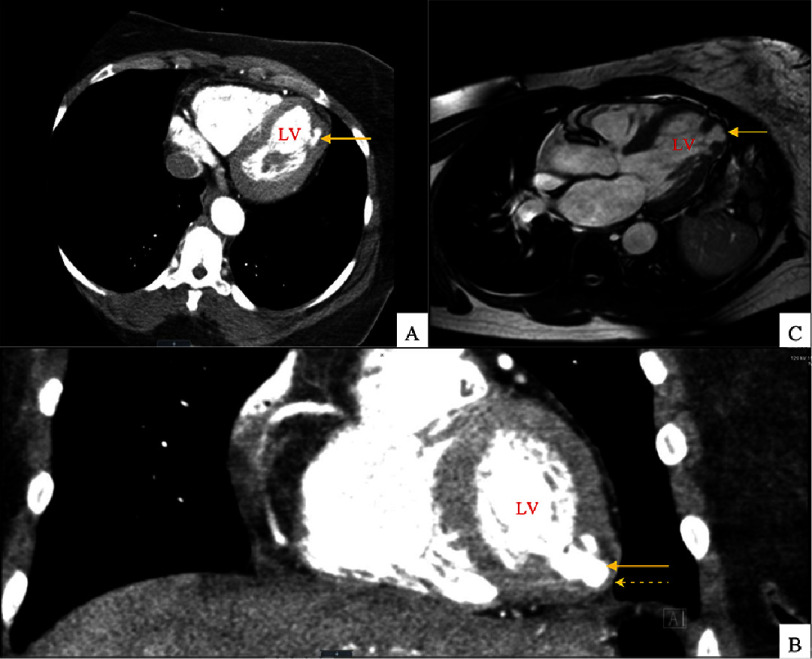
A and B show serial computed tomography (CT) images of a cleft-like deformity at the left ventricular apex (LV), giving rise to a prominent diverticular outpouching that extends laterally from the cleft (solid arrows). The diverticulum maintains continuity with the ventricular cavity through a narrow neck. Figure C shows a T2-weighted cardiac magnetic resonance imaging (MRI) of the outpouching represented as a focal, contractile diverticulum of the apical myocardium (solid arrow). The diverticular wall demonstrates localized myocardial thinning (broken arrow) yet preserves contractility relative to adjacent left ventricular segments.

One year earlier, an exercise stress test showed no ischemic changes, and an echocardiogram revealed no significant structural abnormalities. At the current presentation, her electrocardiogram (ECG) and cardiac troponin levels were normal. Given the persistence of symptoms, an exercise stress echocardiogram was performed, which suggested a possible aneurysm or diverticulum at the left ventricular apex. Cardiac MRI confirmed a focal outpouching at the apex, consistent with a diverticulum, characterized by a thinned myocardium and synchronous contraction with the ventricle. Notably, two papillary muscles appeared displaced apically, forming “hinge points” at the outpouching. A coronary CT angiogram further validated the diagnosis, demonstrating normal coronary arteries and a coronary artery calcium score of zero (Figure 1).

## Discussion

A congenital left ventricular diverticulum is a rare developmental anomaly with an estimated prevalence of 0.42% in adults, based on a large cohort study^1^. While it is more frequently diagnosed in pediatric populations, its presentation in adults is uncommon and often linked to incidental findings or symptomatic manifestations^2^. The condition arises during embryogenesis, around the fourth week, when incomplete ventricular morphogenesis leads to an outpouching of the myocardial wall^3^. Histologically, diverticula typically consist of all three cardiac layers (endocardium, myocardium, and epicardium), distinguishing them from acquired pseudoaneurysms, which lack complete myocardial integrity^4^.

The clinical spectrum of left ventricular diverticula is broad. While many cases remain asymptomatic, symptomatic presentations include systemic thromboembolism (e.g., stroke or transient ischemic attack [TIA]), heart failure, valvular regurgitation, ventricular arrhythmias, wall rupture, or sudden cardiac death^5^. In our patient, recurrent chest pain was the primary symptom, an atypical manifestation potentially related to localized myocardial strain or microvascular dysfunction within the diverticulum. A notable case reported by Jung et al. described a 43-year-old man with recurrent TIAs due to a large apical diverticulum, which was successfully managed with surgical resection via cardiopulmonary bypass^6^. This highlights the potential for serious complications even in previously asymptomatic individuals.

Differentiating a diverticulum from a congenital ventricular aneurysm is critical for management. Diverticula typically feature a narrow neck connecting to the ventricle and contract synchronously with ventricular systole, whereas aneurysms have a wider base and are often akinetic or dyskinetic^7^. In our case, the synchronous contraction observed on CMR supported the diagnosis of a diverticulum over an aneurysm. However, the apical location poses diagnostic challenges, as standard echocardiography may miss subtle abnormalities due to limited acoustic windows at the apex^8^. Advanced imaging, such as CMR, is invaluable, offering high-resolution visualization of myocardial structure, contractility, and tissue characteristics(e.g., absence of scarring or fibrosis), as seen in our patient^9^.

The pathophysiology underlying symptoms like chest pain in diverticula remains poorly understood. Hypotheses include mechanical stress on the thinned myocardium, localized ischemia due to altered coronary microcirculation, or irritation of adjacent pericardial tissue^10^. In our patient, the absence of coronary artery disease (confirmed by CT angiography) and a calcium score of zero suggests that the chest pain was not ischemic in origin but rather a consequence of the diverticulum itself. The displacement of papillary muscles observed on CMR may also contribute, potentially causing subtle mitral valve dysfunction or altered ventricular dynamics, though no regurgitation was noted in this case^11^.

Management of left ventricular diverticula remains controversial and should be tailored to the patient’s clinical profile. Asymptomatic cases discovered incidentally may warrant conservative monitoring with serial imaging to assess for progression or complications^12^. However, symptomatic patients or those with high-risk features (e.g., arrhythmias, thromboembolism) may require intervention. Surgical resection is the gold standard for large or symptomatic diverticula, particularly when associated with systemic embolization or heart failure^13^. For instance, a case series by Ohlow et al. reported successful surgical outcomes in patients with symptomatic diverticula, with reduced morbidity post-resection^5^. Alternatively, anticoagulation is recommended following embolic events, while radiofrequency ablation or implantable cardioverter-defibrillator (ICD) placement may be considered for ventricular tachycardia^14^. Antiarrhythmic drugs, such as amiodarone, have also been used in cases of arrhythmia with variable success^15^.

Our patient’s recurrent chest pain, despite normal coronary arteries and stress testing, underscores the need for heightened suspicion of structural anomalies in atypical presentations. The initial negative stress test likely reflects its limited sensitivity for detecting non-ischemic etiologies, reinforcing the value of advanced imaging like CMR or coronary CT in such cases^16^.

We propose that patients with unexplained chest pain and unremarkable initial workups undergo a stepwise evaluation, potentially including nuclear stress testing, CMR, or CT angiography, to exclude rare entities like ventricular diverticula. This approach aligns with findings from a recent study by Li et al., which emphasized the diagnostic utility of CMR in adult patients with suspected congenital cardiac anomalies^2^.

The long-term prognosis of left ventricular diverticula varies. Asymptomatic cases often have a benign course, but symptomatic patients face elevated risks of complications, with sudden death reported in up to 10% of untreated cases with arrhythmias^17^. In our patient, the absence of high-risk features (e.g., thrombus, arrhythmia) supports a conservative strategy with close follow-up, though her atypical chest pain warrants ongoing investigation into its precise mechanism. She was followed up in the cardiology clinic 3 times after her initial presentation without any worsening of her pain or condition. The decision was made to manage her conservatively with regular imaging follow up including CT and MRI. A surgical intervention will be considered if her pain got worse, developed complications like arrhythmia, embolism or increase in size. Anticoagulation may be also considered if there is a risk of thromboembolism.

## What have we learned?

This case highlights that congenital left ventricular diverticulum, although rare in adults, should be considered in the differential diagnosis of unexplained cardiac symptoms, particularly when initial testing is inconclusive. Advanced imaging modalities, especially cardiac magnetic resonance (CMR), are essential for accurate diagnosis and differentiation from other structural anomalies such as aneurysms. Management must be individualized, balancing conservative monitoring with the potential need for surgical intervention based on symptom progression or complications. Ultimately, a systematic and stepwise diagnostic approach can improve recognition of rare congenital anomalies and guide optimal patient care.

## Declarations

The patient consented for publication of this case study.

The authors disclose that they have no conflict of interests.

## Author statement

Husam Katib collected patient data, conducted the literature review, and drafted the initial manuscript. Nawfal Al Khafagy provided supervision, critical revision, and final approval of the manuscript. Sarah Alem and Manoj Sharma contributed to the imaging analysis and interpretation. Hamza Yousaf contributed to the critical revision of the case details and discussion.
